# A survey on the safety of the SARS-CoV-2 vaccine among a population with stroke risk in China

**DOI:** 10.3389/fmed.2022.859682

**Published:** 2022-09-21

**Authors:** Gang Wu, Meixian Zhang, Xiaomei Xie, Yanwu Zhu, Hongxia Tang, Xinmiao Zhu, Yifan Liang, Tao Chen, Kuangyao Zhu, Danfeng Zhang, Sujun Jiang, Zhengli Jiang, Shaofa Ke

**Affiliations:** ^1^Department of Pharmacy, Taizhou Hospital of Zhejiang Province Affiliated to Wenzhou Medical University, Linhai, China; ^2^Department of Neurology, Taizhou Hospital of Zhejiang Province Affiliated to Wenzhou Medical University, Linhai, China; ^3^Evidence-Based Medicine Center, Taizhou Hospital of Zhejiang Province Affiliated to Wenzhou Medical University, Linhai, China; ^4^Department of Prevention and Health Care, Health Service Center of Gucheng Community, Linhai, China

**Keywords:** SARS-CoV-2 vaccine, safety, stroke risk, adverse reactions, sleep, vaccine knowledge

## Abstract

**Background:**

The safety of the COVID-19 vaccine in patients at stroke risk is poorly understood.

**Methods:**

A survey was conducted on risk factors related to stroke and adverse reactions to vaccines. The participants were divided into low-, medium-, and high-risk groups, according to the stroke risk scorecard recommended by the Stroke Prevention and Control Engineering Committee of the National Health and Family Planning Commission. Factors associated with adverse reactions were analyzed. Reasons for non-vaccination and the aggravation of underlying diseases after vaccination were investigated.

**Results:**

1747 participants participated (138 unvaccinated) and 36.8, 22.1, 41.1% of the vaccinated participants had low, medium, high risk of stroke, respectively. The incidence of adverse reactions after the first and second injection was 16.6, 13.7%, respectively. There was no difference in the incidence of adverse reactions among different risk groups. Sex, vaccine type, sleep quality, worry of adverse reactions, age, and education level were significantly related to adverse reactions to vaccination. The most popular reason for non-vaccination for medium- or high risk-participants was the aggravation of the existing disease. Only 0.3% of vaccinated participants reported slight changes in blood pressure, sugar levels, and lipid levels. No aggravation of stroke sequelae, atrial fibrillation, or transient ischemic attack was reported.

**Conclusions:**

Vaccination against COVID-19 (inactive virus) is safe for people at risk of stroke when the existing disease condition is stable. It is suggested to strengthen vaccine knowledge and ensure good sleep before vaccination.

## Background

The severe acute respiratory syndrome coronavirus 2 (SARS-CoV-2) has a great impact on people's physical and mental health and social life. SARS-CoV-2 not only causes damage to the respiratory system, but also leads to nervous system-related damage, such as loss of sense of smell, memory loss and so on (1). The nerve injury caused by SARS-CoV-2 is related to vascular injury (2). SARS-CoV-2 enters the host cells through Angiotensin-converting enzyme 2 (ACE2) which is abundantly expressed in brain endothelial cells and pericytes, and thereafter causes functional impairment of endothelial cells and pericytes and cerebrovascular disorders (3–7).

In the current lack of specific drugs, vaccination is an effective way to control the COVID-19 pandemic (8). However, sporadic adverse events in the cardiovascular system (9, 10) were reported to occur after SARS-CoV-2 vaccination, such as immune thrombotic thrombocytopenia (11), idiopathic thrombocytopenic purpura, arterial thromboembolic events (such as ischemic stroke), hemorrhagic events (such as hemorrhagic stroke), and cerebral venous sinus thrombosis (12–15). These might increase the hesitation of people with cardiovascular disease or at risk of cardiovascular disease to be vaccinated against COVID-19.

An important goal of the global vaccination campaign is to persuade people to get vaccinated, which will be accelerated by instilling confidence in potential COVID-19 vaccines with safety data (16, 17). Stroke has become the leading cause of death and disability in China and many elderly have high risk of stroke (18), and it needs urgently to know the safety of SARS-CoV-2 vaccine among this population with stroke risk. The purpose of this study was to investigate the safety of the COVID-19 vaccine in people at risk of stroke and guide the implementation of vaccination worldwide.

## Methods

### Study design and population

We conducted the National Stroke Screening Survey on people over 40 years old in a rural village and a urban community in Linhai City, China, to obtain information about risk factors of cardiovascular and cerebrovascular diseases (19). The two areas were chosen according to the proportion to the local population size and geographical locations. Meanwhile, adverse reactions to the COVID-19 vaccine were investigated. The cluster sampling method was used and all residents aged ≥40 years in both two areas were surveyed. The survey was conducted face-to-face at the appointed time by trained investigators, and the participants were asked to answer the questions on the questionnaires. The investigators recorded the answers in the questionnaire, imported the data into MS Excel. The investigators had the same background of cerebrovascular disease. They had been trained on knowledge of COVID-19 vaccine and the standardized procedures, and passed the training examination. Professional quality control personnel supervised the conduction of the research. The survey was conducted between 3 June 2021 and 18 September 2021.

### Questionnaires

The questionnaire was divided into two parts as follows: The investigation of risk factors related to stroke and adverse reactions to the vaccine. The survey of risk factors related to stroke was based on questionnaire of China National Stroke Screening and Prevention Project (20), which included basic demographic information (such as age, sex, education level, occupation, and marital status), lifestyle (e.g., smoking, drinking, exercise, and dietary habits), major medical history (heart disease, hypertension, diabetes, dyslipidemia, etc.), and family history. At the same time, physical examination, ECG examination, and laboratory examination of blood sugar and blood lipids were performed. Laboratory examination results were also imported into MS Excel and used to diagnose emerging diseases, such as heart disease, hypertension, diabetes, and dyslipidemia.

The eight risk factors for stroke included high blood pressure, dyslipidemia, diabetes, smoking, atrial fibrillation or valvular heart disease, obesity, lack of exercise, and family history of stroke. According to the stroke risk scorecard recommended by the Stroke Prevention and Control Engineering Committee of the National Health and Family Planning Commission (21), the population was divided into low-, medium-, and high-risk groups: people with three or more of the above factors or a history of stroke or transient ischemic attack (TIA) were considered to have a high stroke risk. People with one of the three factors (hypertension, diabetes, and atrial fibrillation) were considered to have a medium stroke risk. The rest were considered to have a low stroke risk.

The questionnaire on adverse reactions was based on the vaccine manual and revised according to the advice of preventive experts, which included the following: (1) the producer of the used SARS-CoV-2 vaccine. In Linhai city, the vaccines that had been marketed and used were inactivated vaccines produced by Beijing SINOVAC LIFE Sciences Co., Ltd., Beijing Institute of Biological Products Co., Ltd., Wuhan Institute of Biological Products Co., Ltd., adenoviral vector vaccine produced by CanSino Biologics Inc., and recombinant subunit vaccine produced by Anhui Zhifei Longcom Biopharmaceutical Co., Ltd.; (2) allergy history; (3) the number of doses, local and systemic adverse reactions after each dose; (4) knowledge of vaccine being used (What type of SARS-CoV-2 vaccine were you injected?); (5) attitude toward the SARS-CoV-2 vaccine (“Will you take the SARS-CoV-2 vaccine for your family proactively?” and “Are you worried about the adverse reactions of the SARS-CoV-2 vaccine?”) (22); (6) whether existing diseases were aggravated after vaccination. The reasons for the non-vaccination of the unvaccinated population were also investigated. The questionnaire was included in the Supplementary material.

### Statistical analysis

Categorical variables were expressed as proportions (%) and continuous variables were expressed as the mean ± standard deviation when the data conformed to the normal distribution or median (quartile) when non-normal distribution was observed. Univariate analysis *via* the χ^2^ test was used to assess the potential factors associated with adverse reactions. Multinomial logistic regression was used to identify the factors associated with adverse reactions. Tests were two-sided, with significance set at *P* ≤ 0.05. Data analysis was performed using SPSS software (version 16.0, SPSS Inc.).

## Results

### Demographics and characteristics of the study population

A total of 1,747 (74%, 1,747/2,374) community or village residents over the age of 40 completed the survey. Reasons for non-participation included subjective refusal after knowing the content of the survey, or lack of time to participate in the survey. Of the participants surveyed, 138 were unvaccinated, and 1,609 were vaccinated. Among the vaccinated participants, the age is 59.1 ± 9.5, 1,124 were female (69.9%). Marital status, education level, and occupation are presented in Table 1. Five hundred and ninety-two (36.8%) had a low risk of stroke, 355 (22.1%) had a medium risk of stroke, and 662 (41.1%) had a high risk of stroke. The frequency distribution of stroke risk factors (such as TIA, previous stroke history, hypertension, diabetes) is shown in Table 1. One thousand three hundred and twenty-four participants received the inactive vaccines, 11 received the adenoviral vector vaccine, and 20 received the recombinant subunit vaccine. The rest did not know the vaccine type they received. 81.3% of the participants knew the vaccine being used. 7.8% of the participants worried about adverse reactions to the vaccine, but 98.6% of participants would receive the vaccine for their family and friends. 4.8% had an allergy history (Table 1).

**Table 1:** Baseline characteristics of the vaccinated participants (*n* = 1,609).

**Variables**	**Category**	***n* (%)**
Sex	Male	485 (30.1)
	Female	1,124 (69.9)
Age (years)	40–49	267 (16.6)
	50–59	634 (39.4)
	60–69	461 (28.7)
	70–79	211 (13.1)
	80–90	36 (2.2)
Marital status	Married	1,535 (95.4)
	Others	74 (4.6)
Education level	Primary and below	793 (49.3)
	Junior school	547 (34.0)
	Senior school	211 (13.1)
	College and above	58 (3.6)
Occupation	Mental worker	94 (5.8)
	Business and service personnel	130 (8.1)
	Production personnel in agriculture, forestry, animal husbandry, fishery and water conservancy	750 (46.6)
	Production and transportation equipment operators	163 (10.1)
	Others	472 (29.3)
Risk level	Low risk	592 (36.8)
	Medium risk	355 (22.1)
	High risk	662 (41.1)
Previous TIA	No	1,602 (99.6)
	Yes	7 (0.4)
Previous Stroke	No	1,577 (98.0)
	Yes	32 (2.0)
Family history of stroke	No	1,396 (86.8)
	Yes	213 (13.2)
Aatrial fibrillation or valvular heart disease	No	1,600 (99.4)
	Yes	9 (0.6)
Hypertension	No	743 (46.2)
	Yes	866 (53.8)
Dyslipidemia	No	640 (39.8)
	Yes	969 (60.2)
Diabetes	No	1,352 (84.0)
	Yes	257 (16.0)
Smoking history	No	1,420 (88.3)
	Yes	189 (11.7)
Overweight or obesity	No	1,395 (86.7)
	Yes	214 (13.3)
Lack of exercise	No	673 (41.8)
	Yes	936 (58.2)
Type of vaccine	Inactivated vaccine	1,324 (82.3)
	Adenovirus vector vaccine	11 (0.7)
	Recombinant subunit vaccine	20 (1.2)
	Don't know	254 (15.8)
Knowledge of vaccine being used	No	254 (18.7)
	Yes	1,355 (81.3)
Worry about adverse reactions	No	1,481 (92.2)
	Yes	125 (7.8)
Take vaccine for the family proactively	No	22 (1.4)
	Yes	1,585 (98.6)
Allergic history	No	1,531 (95.2)
	Yes	78 (4.8)

### Adverse reactions in participants with different risk grades of stroke

We analyzed the incidence of adverse reactions in people with a low, moderate, and high risk of stroke after the first and second injection. After the first injection, the incidence of adverse reactions was 18.2, 14.1, and 16.5% in people with low, medium, and high risk of stroke, respectively. The main types of adverse reactions were pain, fatigue at the injection site, and systemic muscle soreness, but there was no difference among the different grades of stroke risk (Table 2). After the second injection, the incidence of adverse reactions was 14.4, 13.7, and 13.3% in people with low, medium, and high risk of stroke, respectively. The main adverse reactions were pain, swelling or itching at the injection site, as well as fatigue, systemic muscle soreness, and rash. There was no difference among the different grades of risk after the second dose (Table 3). The non-solicited adverse reactions include abnormal menstruation, numbness of the limbs, insomnia and palpitations.

**Table 2:** Distribution of multiple types of adverse reactions after first vaccination.

	**Total (*****n*** = **1,609)**			**Low risk (*****n*** = **592)**			**Median risk (*****n*** = **355)**			**High risk (*****n*** = **662)**			
**Adverse reactions**	**No. of subjects**	**Incidence of adverse reactions (%)**	**Proportion of adverse reactions (%)**	**No. of subjects**	**Incidence of adverse reactions (%)**	**Proportion of adverse reactions (%)**	**No. of Subjects**	**Incidence of adverse reactions (%)**	**Proportion of adverse reactions (%)**	**No. of Subjects**	**Incidence of adverse reactions (%)**	**Proportion of adverse reactions (%)**	P*
**Total adverse reactions**	267	16.6	100.0	108	18.2	100.0	50	14.1	100.0	109	16.5	100.0	0.244
**Injection site adverse reactions (pain, induration, redness, swelling or itch)**	123	7.6	46.1	47	8.0	43.5	22	6.2	44.0	54	8.1	49.5	0.503
Pain	96	6.0	36.0	35	5.9	32.4	17	4.8	34.0	44	6.6	40.4	0.501
Induration	10	0.6	3.7	5	0.8	4.6	2	0.6	4.0	3	0.5	2.8	0.664
Redness	9	0.6	3.4	5	0.8	4.6	2	0.6	4.0	2	0.3	1.8	0.426
Swelling or itch	23	1.4	8.6	8	1.4	7.4	5	1.4	10.0	10	1.5	9.2	1.000
**Systemic adverse reactions**	166	10.3	62.2	68	11.5	63.0	30	8.5	60.0	68	10.3	62.4	0.328
Fatigue	51	3.2	19.1	21	3.5	19.4	10	2.8	20.0	20	3	18.3	0.798
Muscle pain	41	2.5	15.4	19	3.2	17.6	8	2.3	16.0	14	2.1	12.8	0.432
Headache	4	0.2	1.5	1	0.2	0.9	1	0.3	2.0	2	0.3	1.8	1.000
Dizziness	25	1.6	9.4	12	2	11.1	5	1.4	10.0	8	1.2	7.3	0.534
Fever	5	0.3	1.9	1	2	0.9	1	3	2.0	3	5	2.8	0.848
Vomiting	0	0.0	0.0	0	0	0.0	0	0	0.0	0	0	0.0	/
Diarrhea	6	0.4	2.2	4	0.7	3.7	1	0.3	2.0	1	0.2	0.9	0.330
Appetite impaired	3	0.2	1.1	1	0.2	0.9	0	0	0.0	2	0.3	1.8	0.799
Nausea	4	0.2	1.5	4	0.7	3.7	0	0	0.0	0	0	0.0	0.054
Cough	0	0.0	0.0	0	0	0.0	0	0	0.0	0	0	0.0	/
Throat pain	6	0.4	2.2	4	0.7	3.7	0	0	0.0	2	0.3	1.8	0.280
Allergic reaction	2	0.1	0.7	1	0.2	0.9	0	0	0.0	1	0.2	0.9	1.000
Urticaria	5	0.3	1.9	0	0	0.0	1	0.3	2.0	4	0.6	3.7	0.136
Rash	26	1.6	9.7	10	1.7	9.3	4	1.1	8.0	12	1.8	11.0	0.718
Stuffy	3	0.2	1.1	2	0.3	1.9	0	0	0.0	1	0.2	0.9	0.612
Runny nose	1	0.1	0.4	1	0.2	0.9	0	0	0.0	0	0	0.0	0.588
Lymphadenopathy	0	0.0	0.0	0	0	0.0	0	0	0.0	0	0	0.0	/
Non-solicited adverse reactions	40	2.5	15.0	19	3.2	17.6	6	1.7	12.0	15	2.3	13.8	0.316

*P-value of the incidence of adverse reactions in three stroke risk grades.

**Table 3:** Distribution of multiple types of adverse reactions after second vaccination.

	**Total (*****n*** = **1,410)**			**Low risk (*****n*** = **523)**			**Median risk (*****n*** = **307)**			**High risk (*****n*** = **580)**			
**Adverse reactions**	**No. of subjects**	**Incidence of adverse reactions (%)**	**Proportion of adverse reactions (%)**	**No. of subjects**	**Incidence of adverse reactions (%)**	**Proportion of adverse reactions (%)**	**No. of subjects**	**Incidence of adverse reactions (%)**	**Proportion of adverse reactions (%)**	**No. of subjects**	**Incidence of adverse reactions (%)**	**Proportion of adverse reactions (%)**	P*
**Total adverse reactions**	193	13.7	100.0	74	14.1	100.0	42	13.7	100.0	77	13.3	100.0	0.910
**Injection site adverse reactions (pain, induration, redness, swelling or itch)**	74	5.2	38.3	28	5.4	37.8	15	4.9	35.7	31	5.3	40.3	0.964
Pain	57	4.0	29.5	22	4.2	29.7	12	3.9	28.6	23	4.0	29.9	0.969
Induration	5	0.4	2.6	1	0.2	1.4	1	0.3	2.4	3	0.5	3.9	0.847
Redness	4	0.3	2.1	3	0.6	4.1	0	0	0.0	1	0.2	1.3	0.357
Swelling or itch	21	1.5	10.9	10	1.9	13.5	4	1.3	9.5	7	1.2	9.1	0.610
**Systemic adverse reactions**	129	9.1	66.8	51	9.8	68.9	32	10.4	76.2	46	7.9	59.7	0.393
Fatigue	27	1.9	14.0	17	3.3	23.0	4	1.3	9.5	6	1	7.8	0.019
Muscle pain	30	2.1	15.5	13	2.5	17.6	8	2.6	19.0	9	1.6	11.7	0.453
Headache	2	0.1	1.0	0	0	0.0	0	0	0.0	2	0.3	2.6	0.354
Dizziness	9	0.6	4.7	2	0.4	2.7	4	1.3	9.5	3	0.5	3.9	0.318
Fever	0	0.0	0.0	0	0	0.0	0	0	0.0	0	0	0.0	/
Vomiting	2	0.1	1.0	0	0	0.0	0	0	0.0	2	0.3	2.6	0.354
Diarrhea	2	0.1	1.0	0	0	0.0	0	0	0.0	2	0.3	2.6	0.354
Appetite impaired	1	0.1	0.5	0	0	0.0	0	0	0.0	1	0.2	1.3	1.000
Nausea	1	0.1	0.5	0	0	0.0	0	0	0.0	1	0.2	1.3	1.000
Cough	0	0.0	0.0	0	0	0.0	0	0	0.0	0	0	0.0	/
Throat pain	3	0.2	1.6	2	0.4	2.7	0	0	0.0	1	0.2	1.3	0.612
Allergic reaction	0	0.0	0.0	0	0	0.0	0	0	0.0	0	0	0.0	/
Urticaria	2	0.1	1.0	1	0.2	1.4	0	0	0.0	1	0.2	1.3	1.000
Rash	29	2.1	15.0	9	1.7	12.2	8	2.6	19.0	12	2.1	15.6	0.696
Stuffy	5	0.4	2.6	1	0.2	1.4	1	0.3	2.4	3	0.5	3.9	0.847
Runny nose	6	0.4	3.1	1	0.2	1.4	1	0.3	2.4	4	0.7	5.2	0.517
Lymphadenopathy	0	0.0	0.0	0	0	0.0	0	0	0.0	0	0	0.0	/
Non-solicited adverse reactions	41	2.9	21.2	16	3.1	21.6	11	3.6	26.2	14	2.4	18.2	0.594

*P-value of the incidence of adverse reactions in three stroke risk grades.

In addition, we investigated whether vaccination aggravated existing diseases, such as atrial fibrillation, hypertension, dyslipidemia, diabetes, stroke sequelae, and frequency of TIA attacks. The results showed that 3 people reported a slight increase in blood pressure, 1 reported a slight increase in blood lipid levels, and 3 reported a slight increase in blood sugar levels. In the vaccinated population, there was no increase in the frequency of TIA attacks and aggravation of stroke sequelae.

### Analysis of factors associated with adverse reactions

To identify the factors associated with adverse reactions, univariate analysis using the χ^2^ test was carried out for participants who were double vaccinated. The factors included sex, age, marital status, education level, occupation, type of vaccine, risk level, previous TIA, previous stroke, family history of stroke, atrial fibrillation or valvular heart disease, hypertension, dyslipidemia, diabetes, smoking history, overweight or obesity, lack of exercise, knowledge of inactivated vaccine being used, worry about adverse reactions, proactive vaccination for the family, and sleep quality before vaccination. The results indicated that sex, age, education level, knowledge of inactivated virus being used, worry about adverse reactions, and sleep quality before vaccination were significantly associated with adverse reactions (Table 4).

**Table 4:** Univariate analysis of factors associated with adverse reactions in completed two doses vaccinated group (*n* = 1,410).

**Variables**	**Categories**	***n***	**Adverse reaction in one vaccination**		**Adverse reaction in both vaccination**		***P***
			***n***	**Frequency (%)**	***n***	**Frequency (%)**	
Total		1,410	232	16.5	78	5.5	
Sex	Male	430	49	11.4	20	4.7	**0.001**
	Female	980	183	18.7	58	5.9	
Age (years)	40–49	230	39	17.0	2222	9.6	**0.001**
	50–59	541	88	16.3	38	7.0	
	60–69	414	76	18.4	8	1.9	
	70–90	225	29	12.9	10	4.4	
Marital status	Married	1,344	227	16.9	74	5.5	0.123
	Others	66	5	7.6	4	6.1	
Education level	Primary and below	710	115	16.2	26	3.7	**0.032**
	Junior school	473	82	17.3	34	7.2	
	Senior school	178	26	14.6	12	6.7	
	College and above	49	9	18.4	6	12.2	
Occupation	Mental worker	82	14	17.1	9	11.0	0.156
	Business and service personnel	114	14	12.3	8	7.0	
	Production personnel in agriculture, forestry, animal husbandry, fishery and water conservancy	673	104	15.5	29	4.3	
	Production and transportation equipment operators and relevant personnel	142	29	20.4	9	6.3	
	Others	399	71	17.8	23	5.8	
Type of vaccine	Inactivated vaccine	1,153	177	15.4	70	6.1	0.336
	Adenovirus vector vaccine	4	1	25.0	1	25.0	
	Recombinant subunit vaccine	19	2	10.5	1	5.3	
Risk level	Low risk	522	86	16.5	35	6.7	0.631
	Medium risk	308	52	16.9	13	4.2	
	High risk	580	94	16.2	30	5.2	
Previous TIA	No	1,404	231	16.5	78	5.6	1.000
	Yes	6	1	16.7	0	0.0	
Previous stroke	No	1,383	224	16.2	77	5.6	0.171
	Yes	27	8	29.6	1	3.7	
Family history of stroke	No	1,231	203	16.5	68	5.5	1.000
	Yes	179	29	16.2	10	5.6	
Atrial fibrillation or	No	1,404	232	16.5	77	5.5	0.237
valvular heart disease	Yes	6	0	0.0	1	16.7	
Hypertension	No	652	111	17.0	44	6.7	0.139
	Yes	758	121	16.0	34	4.5	
Dyslipidemia	No	553	95	17.2	35	6.3	0.461
	Yes	857	137	16.0	43	5.0	
Diabetes	No	1,190	191	16.1	67	5.6	0.623
	Yes	220	41	18.6	11	5.0	
Smoking history	No	1,236	209	16.9	70	5.7	0.359
	Yes	174	23	13.2	8	4.6	
Overweight or obesity	No	1,222	190	15.5	67	5.5	0.057
	Yes	188	42	22.3	11	5.9	
Lack of exercise	No	590	94	15.9	31	5.3	0.830
	Yes	820	138	16.8	47	5.7	
Knowledge of inactivated vaccine being used	No	230	51	22.2	6	2.6	**0.007**
	Yes	1,176	180	15.3	72	6.1	
Worry about adverse reactions	No	1,332	216	16.2	59	4.4	**< 0.001**
	Yes	76	16	21.1	19	25.0	
Take vaccine for the family proactively	No	16	3	18.8	1	6.3	1.000
	Yes	1,393	229	16.4	76	5.5	
Sleep quality before vaccination	Good	816	132	16.2	44	5.4	**< 0.001**
	Moderate	511	74	14.5	23	4.5	
	Poor	58	19	32.8	8	13.8	

The bold values indicated statistically significant difference.

Then, a multinomial logistic regression model was developed to identify the factors associated with adverse effects. Variables that were significant at *P* < 0.05 as a result of the univariate analyses were included. As shown in Table 5, sex [female vs. male, Odds Ratio (OR) = 1.90, 95% confidence interval (CI): 1.33–2.72], knowledge of inactivated vaccine being used (no vs. yes, OR = 1.67, 95% CI: 1.15–2.42), sleep quality before vaccination (good vs. poor, OR = 0.34, 95% CI: 0.18–0.62; moderate vs. poor, OR = 0.29, 95% CI: 0.15–0.55), worry of adverse reactions (no vs. yes, OR = 0.50, 95% CI: 0.27–0.94) were significantly associated with adverse reactions after one vaccination. In addition, age (40–50 vs. ≥70, OR = 1.60, 95% CI: 0.88–2.92), worry of adverse reactions (no vs. yes, OR = 0.12, 95% CI: 0.06–0.24), and sleep quality before vaccination (Good vs. poor, OR = 0.33, 95% CI: 0.13–0.81; moderate vs. poor, OR = 0.18, 95% CI: 0.07–0.47), education level (primary and below vs. college and above, OR = 0.28, 95% CI: 0.1–0.81) were significantly associated with adverse reactions after both vaccinations.

**Table 5:** Multinominal logistic regression of factors associated with adverse reactions in completed two doses vaccinated group (*n* = 1,410).

**Variables**	**Adverse reaction in one vaccination vs. No adverse reaction**			**Adverse reaction in both vaccination vs. No adverse reaction**		
	**OR**			**OR**		
Sex (female vs. male)	1.90	1.33–2.72	**0.000**	1.2289	0.7–2.15	0.471
Knowledge of vaccine being used (no vs. yes)	1.67	1.15–2.42	**0.007**	0.61	0.25–1.48	0.274
Age (years)
40–50 vs. ≥70	1.32	0.73–2.4	0.353	1.60	0.63–4.01	0.321
50–60 vs. ≥70	1.33	0.81–2.19	0.261	1.35	0.6–3.02	0.466
60–70 vs. ≥70	1.53	0.94–2.48	0.086	0.47	0.18–1.26	0.134
Education level
Primary and below vs. College and above	0.65	0.28–1.51	0.319	0.28	0.1–0.81	**0.019**
Junior school vs. College and above	0.93	0.41–2.11	0.860	0.47	0.18–1.26	0.136
Senior school vs. College and above	0.71	0.29–1.74	0.458	0.39	0.13–1.17	0.092
Worry about adverse reactions (no vs. yes)	0.50	0.27–0.94	**0.032**	0.12	0.06–0.24	**0.000**
Sleep quality before vaccination						
Good vs. poor	0.34	0.18–0.62	**0.001**	0.33	0.13–0.81	**0.015**
Moderate vs. poor	0.29	0.15–0.55	**0.000**	0.18	0.07–0.47	**0.000**

The bold values indicated statistically significant difference.

### Reasons for not being vaccinated and the effect of SARS-CoV-2 vaccine on existing diseases

**Figure 1:**
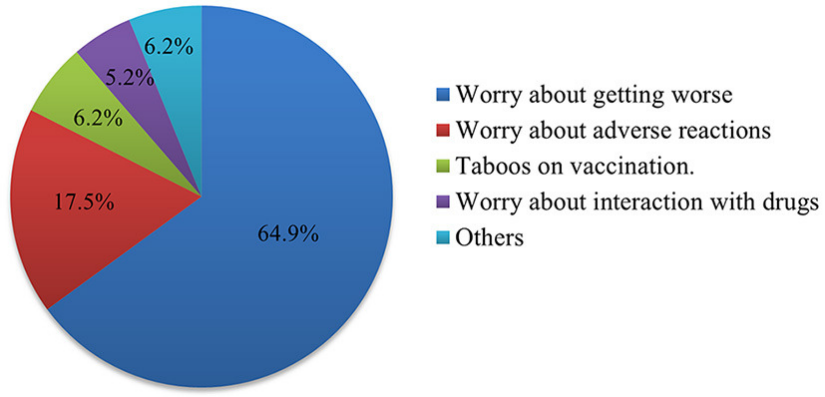
Pie chart showing the reasons why people at medium and high risk of stroke do not want to be vaccinated against COVID-19. The number of each part of the pie chart represents the count and percentage.

In this survey, 138 people were not vaccinated, of whom 120 (87.0%) were at medium or high risk of stroke. We investigated the reasons for not being vaccinated. The results showed that worry about the aggravation of the existing disease was the main cause, with a total of 63 people accounting for 64.9%. Other causes were fear of adverse reactions to the vaccine (17.5%), vaccination taboos (6.2%), and concern about interactions with drugs (5.2%) (Figure 1).

## Discussion

SARS-CoV-2 vaccine is still the effective way to control the pandemic (23). This is a survey study on the safety of the COVID-19 vaccine in a population with stroke risk factors, which guides COVID-19 vaccinations in this population.

We investigated and obtained information on stroke risk factors and adverse reactions of the vaccine in 1747 residents over 40 years old. The results showed that overall, the incidence of adverse reactions after the first injection was 16.6%, and that after the second injection was 13.7%. The main adverse reactions were pain at the injection site, fatigue, systemic soreness, and rash. The relatively low incidence of adverse reactions might be related to the fact that most anticipants (98.0%) were vaccinated with the inactive virus and they showed a lower incidence of adverse reactions than other candidate vaccines (22, 24, 25). However, there was no difference in the incidence of adverse reactions among the different grades of risk after the first or second doses. Hypertension, diabetes mellitus, and cerebrovascular disease have been reported to predispose patients to a more severe outcome of COVID-19 (26). However, the adverse reactions of the COVID-19 vaccine (mainly inactivated vaccine) did not increase with an increase in stroke risk factors.

To identify the factors associated with adverse reactions, univariate analysis was performed first. It was discovered that sex, age, education level, knowledge of inactive virus being used, worry of adverse reactions, and sleep quality before vaccination were associated with adverse reactions for double vaccinated participants. Stroke risk rating and stroke risk factors, such as previous TIA, previous stroke, family history of stroke, atrial fibrillation or valvular heart disease, hypertension, dyslipidemia, diabetes, smoking history, overweight or obesity, and lack of exercise did not show an association with the adverse reaction after completing two doses of vaccination (*P* > 0.05).

However, it is notable that the frequency of adverse reactions upon one vaccination in people with previous stroke events or obesity was 29.6%, and the frequency of adverse reactions upon both vaccinations was the same as observed in people without previous stroke events. In this study, there were 27 participants with previous stroke events who completed two doses of vaccinations. The time between the last cerebrovascular event and the vaccination was 4(10) years, and the mRS was 0(0). Due to the small sample size, the long interval between stroke event and vaccination, and mild neurological impairment of previous stroke events, a more comprehensive investigation needs to be designed to study the relationship between past stroke events and vaccine adverse reactions.

After multinomial logistic regression analysis, it was found that female sex and little knowledge of the vaccine being used was linked to more adverse reactions and less worry of adverse reactions, good sleep before vaccination, and an education level of primary and below were linked to fewer adverse reactions. Therefore, before vaccination, we should strengthen the vaccine type knowledge, ensure a good sleep, and alleviate the worry of adverse reactions. In addition, the potential anxiety states of vaccine recipients might be a contributing factor of adverse reactions, as female, aged around 50 years, fear of adverse reactions, and poor sleep quality are indicative of anxiety states in people about to receive the vaccine. Some psychological interventions are necessary to reduce adverse reactions before vaccination.

The population is aging in the world and one in 11 people (9%) was over 65 in 2019 (27). It is necessary to pay special attention to the vaccination among the elderly population. Some elderly remain reluctant to be vaccinated against COVID-19 and factors influencing vaccination among them included the underlying chronic diseases and polypharmacy (28). Notably, the first cause of not being vaccinated in participants with medium- or high- risk of stroke was the possibility of aggravation of the existing disease. However, the number of people reporting changes in blood pressure, lipid levels, and sugar levels was 3(0.2%), 1(0.06%), 3(0.2%), respectively. For 1609 participants, aggravation of stroke sequelae or TIA attack was not reported. Therefore, the incidence rate of aggravation of the existing disease is very low, and there is no need to worry too much that the vaccine will aggravate the existing condition if the condition is stable.

Since obesity, diabetes, and hypertension and other risk factors of cerebrovascular or cardiovascular disease have been associated with severe outcome of COVID-19 infection, those with relatively higher-risk cardiovascular or stroke conditions should prioritize their receipt of the vaccine (29, 30).

This study has some limitations. First, it is not certain whether the reported adverse events are attributable to vaccination, and the incidence of adverse reactions may be overestimated. Second, the sample size of previous stroke events is small; therefore, it is impossible to determine the relationship between previous stroke type, infarction size, last onset time, mRS score, and vaccine adverse reactions, which requires further investigation. Third, as this is a survey study, bias cannot be avoided due to the presence of subjective factors, although we have taken many measures to reduce it.

## Conclusions

For people at risk of stroke, vaccination against COVID-19 (inactive virus) is safe when the existing disease condition is stable and potentially reduces the risk of infection or critical illness. Age, sex, level of awareness of vaccine, worry of adverse reactions to the vaccine, and education level are related to adverse reactions after vaccination. It is suggested to strengthen vaccine knowledge and ensure good sleep before vaccination. This positive evidence for the safety of the vaccine (inactivated vaccine) may help to enhance the vaccination rate and provide guidelines for the implementation of vaccination among people at stroke risk in the future.

## Data availability statement

The original contributions presented in the study are included in the article/Supplementary material, further inquiries can be directed to the corresponding author/s.

## Ethics statement

The studies involving human participants were reviewed and approved by Ethics Committee of Taizhou Hospital of Zhejiang Province. The patients/participants provided their written informed consent to participate in this study.

## Author contributions

Conceptualization: GW, SK, and ZJ. Investigation: XX, SJ, YZ, HT, XZ, YL, TC, KZ, and DZ. Formal analysis and writing—original draft: GW and MZ. Writing—review and editing and resources: SK and ZJ. All authors reviewed the manuscript, contributed to the article, and approved the submitted version.

## Funding

This study was in part supported by National Natural Science Foundations of China (No. 81903584) and Zhejiang Provincial Basic and Public Welfare Research Program (LGD20H310002 to GW).

## Conflict of interest

The authors declare that the research was conducted in the absence of any commercial or financial relationships that could be construed as a potential conflict of interest.

## Publisher's note

All claims expressed in this article are solely those of the authors and do not necessarily represent those of their affiliated organizations, or those of the publisher, the editors and the reviewers. Any product that may be evaluated in this article, or claim that may be made by its manufacturer, is not guaranteed or endorsed by the publisher.
