# Supplementary material for: A survey on the safety of the SARS-CoV-2 vaccine among a population with stroke risk in China
Source: Front Med (Lausanne). 2022 Sep 21;9:859682. doi: 10.3389/fmed.2022.859682 (PMC9532547; doi:10.3389/fmed.2022.859682)
Supplement: Supplementary file 1 [file Table_1.DOCX]

**Questionnaire on the safety of the SARS-CoV-2 vaccine among a population with stroke risk**

This is a questionnaire on adverse reactions of the COVID-19 vaccine and the possible influencing factors. The data collected were only used for statistical analysis. This study was approved by the Ethics Committee. You are volunteered to participate in this survey.

Please tick (√) before correct option

**Have you been vaccinated with COVID-19 vaccine?**

□Yes □No

**If you are vaccinated, answer the following questions:**

1. Which COVID-19 vaccine did you receive?

□ Beijing Shengwu □ Beijing Kexingzhongwei □ Wuhan □ Kangxinuo □ Anhui Zhifeilongkema

2. What is the type of COVID-19 vaccine you reveived?

□ RNA vaccine □ Inactivated vaccine □ Viral vector vaccine

□ Protein/virus-like particle vaccine □ Don’t know

3. If conditions permit, will you take the COVID-19 vaccine for your family proactively?

- Yes □ No

4. Are you concerned about adverse effects of the COVID-19 vaccine?

- Yes □ No

5. Sleep quality before vaccination with the COVID-19 vaccine:

- Good □ Moderate □ Bad

6. Have you had any adverse reactions to other vaccines?

- Yes □ No

7. Are you allergic to the following items (check all that apply) :

- Dust mites □ food (such as eggs, peanuts, seafood, mango et al.) □ pollen □ alcohol □ penicillin or other drugs □ others

8. How many doses of the COVID-19 vaccine have you been vaccinated with?

- 1 dose □ 2 doses

9. Did you experience the following adverse reactions after receiving the first and second doses of the COVID-19 vaccine?

- Yes □ No

Please tick the corresponding adverse reaction：

| Adverse reactions | After first dose | After second dose |
| --- | --- | --- |
| Injection site adverse reactions | | |
| Pain | □ | □ |
| Induration | □ | □ |
| Redness | □ | □ |
| Swelling or itch | □ | □ |
| Systemic adverse reactions | | |
| Fatigue | □ | □ |
| Muscle pain | □ | □ |
| Headache | □ | □ |
| Dizziness | □ | □ |
| Fever | □ | □ |
| Vomiting | □ | □ |
| Diarrhoea | □ | □ |
| Appetite impaired | □ | □ |
| Nausea | □ | □ |
| Cough | □ | □ |
| Throat pain | □ | □ |
| Allergic reaction | □ | □ |
| Urticaria | □ | □ |
| Rash | □ | □ |
| Stuffy | □ | □ |
| Runny nose | □ | □ |
| Lymphadenopathy | □ | □ |
| Other | □ | □ |
|  |  |  |
|  |  |  |
|  |  |  |
|  |  |  |

10. After vaccination with COVID-19 vaccine,

□ Atrial fibrillation more frequent □ Blood pressure increased □ Lipid elevated □ Blood glucose increased □ Worsening of stroke sequelae □ TIA more frequent

**If you are not vaccinated, answer the following questions (check all that apply) :**

1. Reasons for not being vaccinated:

- Worry about the existing disease getting worse
- Worry about adverse reactions of the COVID-19 vaccine

□ Taboos on vaccination.

□ Worry about interaction with the drugs already in use

□ Others
